# Increased circulating endothelial progenitor cells and improved short-term outcomes in acute non-cardioembolic stroke after hyperbaric oxygen therapy

**DOI:** 10.1186/s12967-018-1629-x

**Published:** 2018-09-12

**Authors:** Chen-Yu Chen, Re-Wen Wu, Nai-Wen Tsai, Mel S. Lee, Wei-Che Lin, Mei-Chi Hsu, Chih-Cheng Huang, Yun-Ru Lai, Chia-Te Kung, Hung-Chen Wang, Yu-Jih Su, Chih-Min Su, Sheng-Yuan Hsiao, Ben-Chung Cheng, Yi-Fang Chiang, Cheng-Hsien Lu

**Affiliations:** 1 Department of Hyperbaric Oxygen Therapy Center, Kaohsiung Chang Gung Memorial Hospital, Chang Gung University College of Medicine, Kaohsiung, Taiwan; 2 Department of Orthopaedic Surgery, Kaohsiung Chang Gung Memorial Hospital, Chang Gung University College of Medicine, Kaohsiung, Taiwan; 3 Department of Neurology, Kaohsiung Chang Gung Memorial Hospital, Chang Gung University College of Medicine, Kaohsiung, Taiwan; 4 Department of Radiology, Kaohsiung Chang Gung Memorial Hospital, Chang Gung University College of Medicine, Kaohsiung, Taiwan; 5 Department of Nursing, I-Shou University, Kaohsiung, Taiwan; 6 Department of Emergency Medicine, Kaohsiung Chang Gung Memorial Hospital, Chang Gung University College of Medicine, Kaohsiung, Taiwan; 7 Department of Neurosurgery, Kaohsiung Chang Gung Memorial Hospital, Chang Gung University College of Medicine, Kaohsiung, Taiwan; 8 Department of Rheumatology, Allergy and Immunology, Kaohsiung Chang Gung Memorial Hospital, Chang Gung University College of Medicine, Kaohsiung, Taiwan; 9 Department of Nephrology, Kaohsiung Chang Gung Memorial Hospital, Chang Gung University College of Medicine, Kaohsiung, Taiwan; 10 Department of Biological Science, National Sun Yat-Sen University, Kaohsiung, Taiwan; 11 Department of Neurology, Xiamen Chang Gung Memorial Hospital, Xiamen, Fujian China

**Keywords:** Endothelial progenitor cells, Hyperbaric oxygen therapy, Non-cardioembolic stroke, National Institutes of Health Stroke Scale, Barthel index, Modified Rankin Scale

## Abstract

**Background:**

Acute ischemic stroke is a leading cause of mortality and long-term disability, and profiles of endothelial progenitor cells (EPCs) reflect the degree of endothelial impairment. This study tested the hypothesis that hyperbaric oxygen therapy (HBOT) both improves the clinical short-term outcomes and increases the number of circulating EPCs and antioxidant capacity.

**Methods:**

The numbers of circulating EPCs [CD133^+^/CD34^+^ (%), KDR^+^/CD34^+^ (%)], biomarkers for oxidative stress (thiols and thiobarbituric acid-reactive substances), and clinical scores (National Institutes of Health Stroke Scale [NIHSS], Barthel index [BI], and modified Rankin Scale [MRS]) were prospectively evaluated in 25 patients with acute non-cardioembolic stroke under HBOT at two time points (pre- and post-HBOT). The biomarkers and clinical scores were compared with those of 25 age- and sex-matched disease controls.

**Results:**

The numbers of KDR^+^/CD34^+^ (%) in the HBOT group following HBOT increased significantly, whereas the numbers of CD133^+^/CD34^+^ (%) also showed a tendency to increase without statistical significance. The mean high-sensitivity C-reactive protein levels showed significant decrease post-HBOT follow-up in the HBOT group. The changes in KDR^+^/CD34^+^EPC (%) numbers were positively correlated with changes in clinical outcomes scores (BI, NIHSS, and MRS) in the HBOT group.

**Conclusions:**

Based on the results of our study, HBOT can both improve short-term clinical outcomes and increase the number of circulating EPCs in patients with acute non-cardioembolic stroke.

## Background

Acute ischemic stroke is a leading cause of mortality, long-term disability, and neurological sequelae worldwide [1]. After acute cerebral ischemia, a complicated cascade of biochemical events occurs. This phenomenon involves inflammation, an increased production of free radicals and reactive oxygen species (ROS) in the tissue and plasma, increased platelet activation and platelet–leukocyte interactions, inflammatory cell adhesion molecule production, firm adhesion, and transmigration of leukocytes along the vessel wall. These events finally contribute to endothelial and neurological dysfunction [2, 3].

Endothelial progenitor cells (EPCs) are a subpopulation of bone marrow mononuclear cells that are capable of generating new blood vessels in areas of ischemia or infarction and serve as a circulating pool of cells to replace dysfunctional endothelium [4–6]. EPCs are exposed to oxidative stress during vascular injury, and an increase in circulating EPCs after acute ischemic stroke is associated with a good outcome [7, 8].

Hyperbaric oxygen therapy (HBOT), which combines the action of hyperoxia and hyperbaric pressure, leads to significantly improved tissue oxygenation and could restore neuronal activity in metabolically dysfunctional areas and is an adjunctive therapy for the treatment of patients with ischemic stroke [9]. The potential mechanisms of HBOT include improving cerebral blood flow, initiating vascular repair, inducing regeneration of axonal white matter, restoring the functional blood–brain barrier, and reducing inflammatory reactions and brain edema. These events finally can activate neuroplasticity and revitalize chronically impaired brain functions in the metabolically dysfunctional stunned areas [10].

Ischemia/reperfusion injury after ischemic stroke has been recognized as the most frequent cause of devastating disorders and death. Mitochondria operate aberrantly in response to ischemia, and the sudden delivery of oxygen back to previously ischemic cells can generate additional free radicals (reperfusion injury). HBOT improves oxygen delivery to the mitochondria (which is mostly affected by the oxygen gradient resulting from the dissolved oxygen) as well as mitochondrial function, reducing the amount of unwanted mitochondrial by-products such as ROS, alleviating oxidative stress, reducing apoptosis and inflammatory effects, and reducing ischemia–reperfusion injury by inhibiting apoptosis via the mitochondrial pathway [11, 12].

With regard to the research on the efficacy of HBOT in patients with acute ischemic stroke, comparative evidence of its effectiveness is limited. Most previous studies had various methodological qualities, including selection, performance, detection and attrition bias (incomplete outcome data) [13], and the lack of a functional score for outcome prediction or a magnetic resonance imaging (MRI)-based study of the diagnosis of acute ischemic stroke [13–17]. To date, information on the effects of HBOT for patients with acute ischemic stroke in terms of clinical efficiency and biomarkers of endothelial dysfunction is scarce. This study tested the hypothesis that HBOT not only improves clinical short-term outcomes, it increases the number of circulating EPCs and antioxidant capacity. The successful clinical translation of these approaches has the potential to increase our understanding of the mechanism and improve the quality of life of patients with acute ischemic stroke.

## Methods

### Study design

This single-center prospective case–control study was conducted at Chang Gung Memorial Hospital-Kaohsiung, a medical center and the main referral hospital serving an area with 3 million inhabitants in southern Taiwan.

### Diagnostic criteria of acute ischemic stroke

In this study, acute ischemic stroke was defined as an acute-onset loss of focal cerebral function persisting for at least 24 h as well as results of brain MRI with diffusion-weighted imaging within 1 week of the event.

### Inclusion and exclusion criteria

Based on the clinical evaluation and imaging study results, patients with non-cardioembolic ischemic stroke, including large- and small-artery diseases, two stroke subtypes, were enrolled in this study according to the Trial of ORG 10172 in Acute Stroke Treatment criteria [18]. We enrolled 25 patients with first-event acute non-cardioembolic stroke undergoing HBOT. For comparison, 25 age-, sex-, and body mass index (BMI)-matched patients with acute non-cardioembolic stroke who did not undergo HBOT were included as disease controls. The hospital’s Institutional Review Committee on Human Research approved the study protocol (104-2149B), and all enrolled patients provided full informed written consent.

Patients with any of the following were excluded from this study: (1) cardioembolic stroke, congestive heart failure, and a history of atrial fibrillation or valvular heart disease; (2) evidence of fever after stroke or a history of infection 1 week before the stroke; (3) intracranial hemorrhage or a history of recent chest or ear surgery within the preceding 3 months; and (4) underlying neoplasm, seizure, or pneumothorax.

Therapeutic regimens for non-cardioembolic ischemic stroke were dependent on American Heart Association/American Stroke Association guidelines [19]. In this study, the patients were classified into the HBOT or disease control group based on their preferences. The HBOT group consisted of patients who underwent HBOT combined with anti-platelet therapy, whereas the disease control group comprised of patients undergoing anti-platelet therapy alone.

### Hyperbaric oxygen therapy

The HBOT patients were placed in a chamber that was pressurized with air to 2.5 ATA during 15 min and were supplied 100% oxygen for 25 min, followed by a 5-min air break. This cycle was repeated once and followed by 100% oxygen for 10 min, after which time the chamber was depressurized to 1 ATA over 15 min with 100% oxygen for a total treatment time of 100 min.

### Biochemical analysis

Blood samples were obtained by antecubital vein puncture in a fasting non-sedative state between 09:00 A.M. and 10:00 A.M. in the control and study groups to exclude the possible influence of circadian variations and were analyzed at the hospital’s central laboratory. Serum levels of triglycerides, total cholesterol, high-density lipoprotein cholesterol (HDL-C), low-density lipoprotein cholesterol (LDL-C), blood sugar, HBA1c, and high-sensitivity C-reactive protein (hs-CRP) were determined.

#### Biomarkers for oxidative stress and anti-oxidative defense

The blood samples were centrifuged at 3000 rpm for 10 min. Each serum sample was collected and frozen at − 80 °C before biochemical measurement. Measurement of thiobarbituric acid-reactive substances (TBARS) is a well-established method for detecting lipid peroxidation, while the ability of anti-oxidative defense in response to increased oxidative damage was evaluated by measuring the serum level of total reduced thiols, a physiological free radical scavenger. The detailed methods were based on our previous study [20].

### Assessment of circulating EPC level

To assess the circulating EPCs, blood samples were collected at baseline (before HBOT) and at 1 month post-HBOT. Peripheral blood EPC levels were determined by measuring the EPC surface markers of CD45/CD34/CD133 and CD45/CD34/KDR and measuring mononuclear cells by flow cytometry. Isotype identical antibodies served as controls (Becton–Dickinson). The detailed methods were also based on our previous study [20].

### Clinical assessment

All subjects underwent complete neurological examinations upon enrollment (Day 4 after stroke) and during the follow-up period (Day 30 post-stroke). Brain MRI with magnetic resonance angiography, a duplex ultrasound study of the carotid arteries, and trans-cranial color-code sonography were performed in the acute phase of the ischemic stroke in the HBOT and disease control groups.

Vascular risk factors included the following: hypertension, currently taking anti-hypertensive drugs, or a blood pressure > 140/90 mmHg at two readings; diabetes mellitus, currently taking anti-diabetic drugs, or an elevated hemoglobin A1c or elevated blood glucose at two readings; and dyslipidemia, currently taking lipid-lowering medication, or total cholesterol > 200 mg/dL or triglycerides > 180 mg/dL [21].

An experienced neurology nurse specialist (Chen-Yu Chen) who was blinded to the patients’ clinical and biochemical data was trained to measure these functional scores at enrollment and the end of the study. Stroke severity was assessed using the National Institutes of Health Stroke Scale (NIHSS). The physical disabilities and handicaps of the stroke patients were evaluated using the Barthel index (BI) and modified Rankin Scale (MRS) during the acute and convalescent phases. Therapeutic outcomes were evaluated before and after HBOT in both patient groups.

### Statistical analysis

Three separate statistical analyses were performed. Categorical variables were compared using the Chi square test or Fisher’s exact test. Continuous variables within the two groups were compared using the independent *t* test and the Mann–Whitney U test for parametric and non-parametric data, respectively. First, demographic data between the HBOT and disease control groups were compared. Second, the changes of each parameter over 1 month were defined as data at 1 month follow-up minus baseline data, and a correlation analysis was used to determine the relationship between changes in EPCs on the changes in biomarkers, including oxidative stress, peripheral blood testing, and functional scores. Third, changes between baseline and 1 month post-HBOT biomarker values and functional score parameters were compared using a paired t-test. Furthermore, repeated-measures analysis of variance was used to compare biomarkers and functional scores at two different time points (enrollment and 1 month after HBOT). Analysis of covariance (ANCOVA) was used to compare subgroups (HBOT and disease control) after the control for potential confounding variables. The Levene test of equality of error variance was used to ensure equal variance in both groups. ANCOVA was used to compare the biomarkers between the subgroups with sex and age as potential confounding variables. All statistical analyses were conducted using SAS software version 9.1 (SAS Statistical Institute, Cary, NC, USA).

## Results

### Patients’ baseline characteristics

The baseline characteristics and laboratory data of the 25 adult HBOT and 25 disease controls revealed that the two groups were similar in terms of age (*p *= 0.679), sex (*p *= 0.774), and BMI (*p *= 0.455) (Table 1). The mean systolic blood pressure and mean diastolic blood pressure did not differ significantly between the HBOT and disease control groups (*p *= 0.202 and *p *= 0.102, respectively). The other peripheral blood studies, including HDL-C, LDL-C, HbA1c, triglyceride, and hs-CRP levels were similar between the two groups, except for calcium level, which was significantly higher in the HBOT group (*p *= 0.041).

**Table 1:** Baseline characteristics and laboratory data of the patients and controls

	HBOT group (n = 25)	Disease controls (n = 25)	p value
Age, years	61.3 ± 8.7	62.7 ± 12.5	0.679
Sex (female:male)	15:10	14:11	0.774
Body mass index	24.7 ± 2.9	23.9 ± 4.1	0.455
NIHSS at enrollment	7.4 ± 3.2	6.4 ± 2.5	0.301
Mean systolic blood pressure (mmHg)	*142.2 *±* 12.1*	*149.7 *±* 25.5*	0.202
Mean diastolic blood pressure (mmHg)	*89.5 *±* 11.6*	*82.2 *±* 18.4*	0.102
Underlying diseases			
Hypertension, n (%)	14 (56%)	11 (44%)	0.396
Diabetes mellitus, n (%)	11 (50%)	11 (50%)	1.0
Dyslipidemia, n (%)	3 (38%)	5 (62%)	0.44
Peripheral blood studies			
White blood cells (×10^3^/mL)	*8.0 *±* 2.2*	*7.9 *±* 2.3*	0.882
Hemoglobin	*14.3 *±* 1.72*	*12.8 *±* 1.6*	0.008
Hematocrit	*42.2 *±* 4.8*	*38.7 *±* 4.5*	0.019
APTT	*27.7 *±* 2.5*	*26.9 *±* 3.5*	0.397
PT	*10.5 *±* 0.5*	*10.3 *±* 0.6*	0.262
Platelet count (×10^4^/mL)	*236.7 *±* 61.4*	*242.8 *±* 45.2*	0.718
Total cholesterol, mg/dL	*207.3 *±* 73.1*	*171.6 *±* 44.0*	0.062
HDL-C, mg/dL	*44.0 *±* 15.0*	*42.6 *±* 12.7*	0.749
LDL-C, mg/dL	*126.5 *±* 64.2*	*99.5 *±* 39.5*	0.108
Triglyceride, mg/dL	*147.4 *±* 72.0*	*132.9 *±* 94.7*	0.58
HbA1c	*8.2 *±* 2.6*	*7.3 *±* 2.1*	0.250
Ca	*9.3 *±* 0.43*	*9.0 *±* 0.44*	0.041
Hs-CRP, mg/L	*5.1 *±* 3.2*	*4.1 *±* 2.8*	0.428

Values are expressed as mean ± SD unless otherwise indicated

*HBOT* hyperbaric oxygen therapy, *HBA1c* glycosylated hemoglobin, *aPTT* activated partial thromboplastin time, *PT* prothrombin time, *hs-CRP* high-sensitivity C-reactive protein, *NIHSS* National Institutes of Health Stroke Scale

### Effect of EPC level changes on changes in peripheral blood testing, oxidative stress biomarkers, and functional outcomes

The changes of each parameter during 1 month defined as data in 1-month follow-up minus baseline data and correlation analysis were used to determine the relationship between EPC changes and changes in biomarkers, including oxidative stress, peripheral blood testing, and functional scores in patients who underwent HBOT. The statistically significant results (correlation coefficient, *p* value) of KDR^+^/CD34^+^EPCs (%) were those of thiols (r = 0.604, *p *=0–0.22), NIHSS (r = − 0.695, *p *=0.002), BI (r = 0.676, *p *= 0.003), and MRS (r = -0.642, *p *= 0.006). The results showed that the increase in KDR^+^/CD34^+^EPCs (%) was positively correlated with an increase in both thiols level and BI score, while the increase in KDR^+^/CD34^+^EPCs (%) was negatively correlated with the decreases in NIHSS and MRS score (Table 2).

**Table 2:** Correlation among EPCs, peripheral blood testing, biomarkers of oxidative stress, and functional score of the patients who underwent hyperbaric oxygen therapy during the study period

Spearman correlation	ΔCD133^+^/CD34^+^EPCs (%)		ΔKDR^+^/CD34^+^EPC (%)	
	r	*p*	r	*p*
ΔTBARS, μmol/L	− 0.201	0.456	− 0.288	0.279
ΔThiols, μmol/L	0.291	0.312	0.604	0.022*
Δhs-CRP, mg/L	− 0.069	0.813	− 0.573	0.032*
ΔsL-selectin (ng/mL)	0.039	0.886	− 0.212	0.430
ΔsP-selectin (ng/mL)	− 0.002	0.996	− 0.156	0.564
ΔsE-selectin (ng/mL)	− 0.006	0.983	− 0.594	0.015*
ΔsICAM-1 (ng/mL)	0.108	0.692	− 0.290	0.275
ΔsVCAM-1 (ng/mL)	− 0.236	0.379	− 0.031	0.910
ΔNIH Stroke Scale	0.061	0.816	− 0.695	0.002***
ΔBarthel index	− 0.111	0.67	0.676	0.003***
ΔModified Rankin Scale	− 0.144	0.582	− 0.642	0.006**

Δ: Mean changes during 1 month (1-month follow-up minus baseline data)

*HbA1c* hemoglobin A1c, *EPC* endothelial progenitor cells, *NIHSS* National Institutes of Health Stroke Scale, *BI* Barthel index, *MRS* modified Rankin Scale, *sL-selectin* soluble leukocyte selectin, *sP-selectin* soluble platelet selectin, *sE-selectin* soluble endothelial selectin, *sICAM* soluble intercellular adhesion molecule, *sVCAM-1* vascular cell adhesion molecule-1

* p < 0.05; ** p < 0.01; *** p < 0.005

### Comparison of baseline characteristics and biomarkers between groups

The baseline biomarkers and functional scores in the HBOT and disease control groups (Table 3) showed that the two groups were similar in terms of hs-CRP (*p *= 0.428), endothelial cell activation markers, including sL-selectin (*p* = 0.706), sP-selectin (*p* = 0.231), sE-selectin (*p* = 0.441), sICAM-1 (*p* = 0.617), sVCAM-1 (*p* = 0.438), TBARS (*p *= 0.339), thiols (*p *= 0.273), NIHSS (*p* = 0.301), BI (*p* = 0.951), and MRS (*p* = 0.881). However, the trends for CD133^+^/CD34^+^ (%) (*p *= 0.555) and KDR^+^/CD34^+^ (%) (*p *= 0.103) was not significantly higher in the disease control group.

**Table 3:** Changes of biomarkers and functional score in the study and control groups during the study period

	HBOT group (n = 25)		Disease control group (n = 25)	
	Baseline	Follow-up	Baseline	Follow-up
Biomarkers of EPC				
CD133^+^/CD34^+^ (%)	28.4 ± 14.4	32.6 ± 15.3	31.2 ± 15.2	25.3 ± 17.9
KDR^+^/CD34^+^ (%)	3.5 ± 1.8	5.6 ± 4.0*	5.1 ± 2.9	4.9 ± 3.4
Biomarkers of oxidative stress				
TBARS, μmol/L	16.9 ± 6.6	14.8 ± 4.57	15.0 ± 5.9	12.2 ± 2.8
Thiols, μmol/L	0.74 ± 0.3	0.81 ± 0.36	0.89 ± 0.39	0.99 ± 0.25
Peripheral blood studies				
Hs-CRP, mg/L	5.1 ± 3.2	3.4 ± 2.1*	4.1 ± 2.8	3.7 ± 2.4
Endothelial cell activation markers				
sL-selectin (ng/mL)	691.4 ± 118.4	689.1 ± 106.0	684.6 ± 184.3	706.4 ± 197.8
sP-selectin (ng/mL)	91.3 ± 18.3	90.6 ± 18.2	84.9 ± 18.4	82.5 ± 15.0
sE-selectin (ng/mL)	48.8 ± 18.6	56.1 ± 37.0	41.4 ± 18.8	42.0 ± 12.0
sICAM-1 (ng/mL)	198.8 ± 64.0	213.7 ± 70.4	217.6 ± 46.5	865.2 ± 289.3
sVCAM-1 (ng/mL)	876.0 ± 302.8	804.3 ± 208.3	865.2 ± 289.3	814.3 ± 173.5
Functional score				
NIHSS	7.4 ± 3.2	4.1 ± 3.5*****	6.4 ± 2.5	4.7 ± 2.7***
Barthel index	40.0 ± 26.1	67.1 ± 29.4*****	40.5 ± 27.5	55.0 ± 29.2*
Modified Rankin Scale	3.3 ± 1.1	2.3 ± 1.5*****	3.4 ± 1.2	2.9 ± 1.3*

Values are expressed as mean ± SD unless otherwise indicated

The changes (baseline and 1-month follow-up) of biomarkers and functional score study in different groups (HBOT and disease control) were compared using paired-t test

*HBOT* hyperbaric oxygen therapy, *sL-selectin* soluble leukocyte selectin, *sP-selectin* soluble platelet selectin, *sE-selectin* soluble endothelial selectin, *sICAM* soluble intercellular adhesion molecule, *sVCAM-1* vascular cell adhesion molecule-1, *NIHSS* National Institutes of Health Stroke Scale

* p < 0.05; ** p < 0.01; *** p < 0.005; **** p < 0.001; ***** p < 0.0001

### Changes in biomarker pre- and post-HBOT in the HBOT and disease control groups

**Fig. 1:**
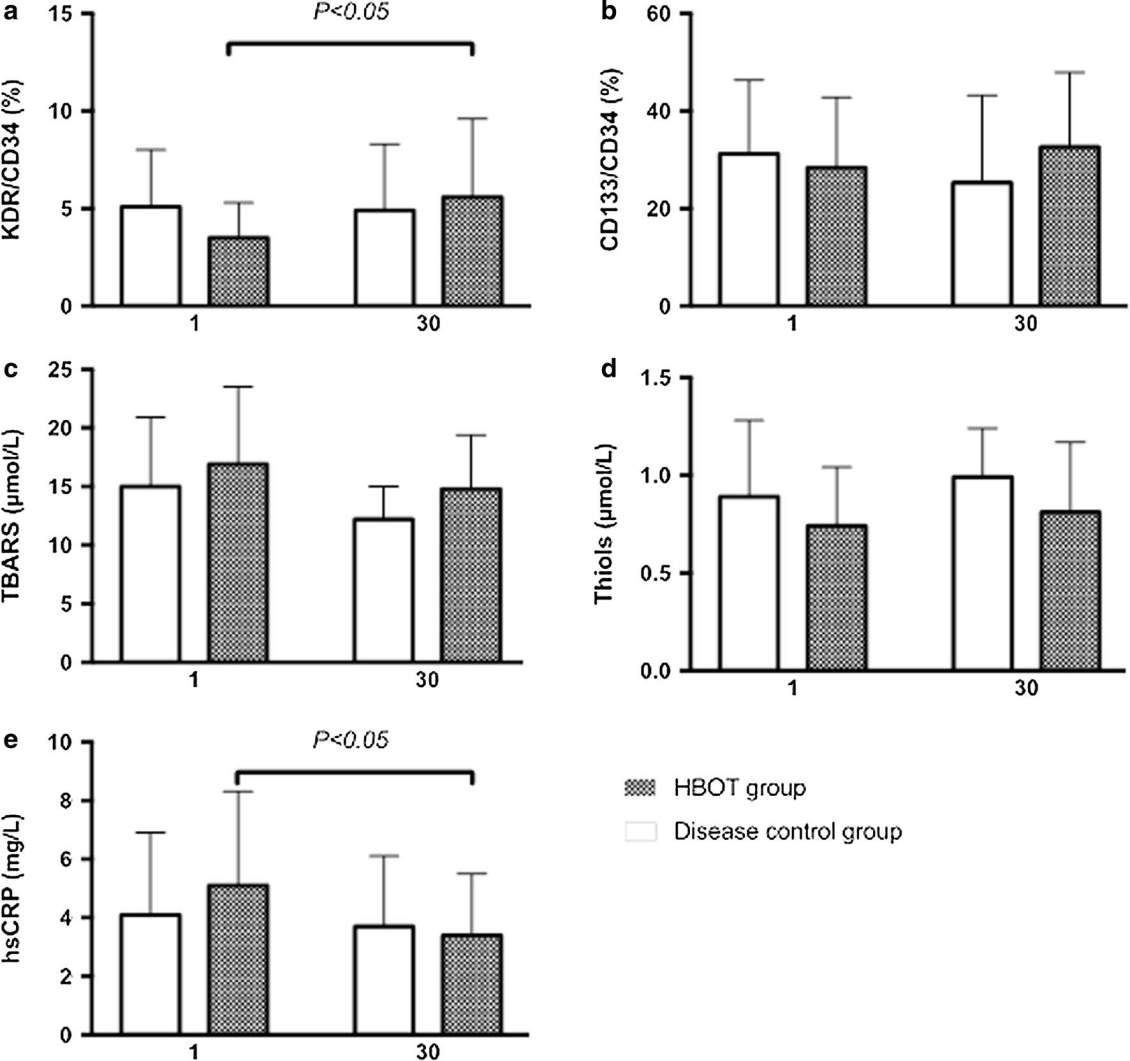
Changes (baseline and one-month follow-up) in biomarker levels in different patient subgroups (HBOT group and disease control) were compared using the paired-t test. **a** KDR^+^/CD34^+^ (%), **b** CD133^+^/CD34^+^ (%), **c** TBARS, **d** thiol, **e** hs-CRP

Changes in biomarkers of biochemical data, oxidative stress, EPC level, and functional scores pre- and post-HBOT (Table 3) showed that the numbers of KDR^+^/CD34^+^ (%) in the HBOT group post-HBOT significantly increased, whereas the numbers of CD133^+^/CD34^+^ (%) also showed a tendency to increase but without statistical significance. The CD133^+^/CD34^+^ (%) and KDR^+^/CD34^+^ (%) numbers tended to decrease without statistical significance during the study period (pre- and post-HBOT). The mean hs-CRP levels showed significantly decreased post-HBOT follow-up in the HBOT group (Fig. 1). The mean levels of sL-selectin, sP-selectin, sE-selectin, sICAM-1, and sVCAM-1 did not show significant changes at 1-month follow-up in either group. The mean TBARS level was decreased, whereas the mean thiol level was increased at 1-month follow-up in both groups, although the changes did not reach statistical significance. To exclude the possible effects of sex and age on the EPC biomarkers, the hypothesis that EPC level was equal between sexes and among ages was tested using ANCOVA. Univariate analysis of covariance between the two treated groups at the two different time points (pre- and post-HBOT in the HBOT group) showed that both CD133^+^/CD34^+^ (%) (*p *= 0.404) and KDR^+^/CD34^+^ (*p *= 0.775) did not differ significantly.

### Changes in clinical score pre- and post-HBOT in the HBOT and disease control groups

**Fig. 2:**
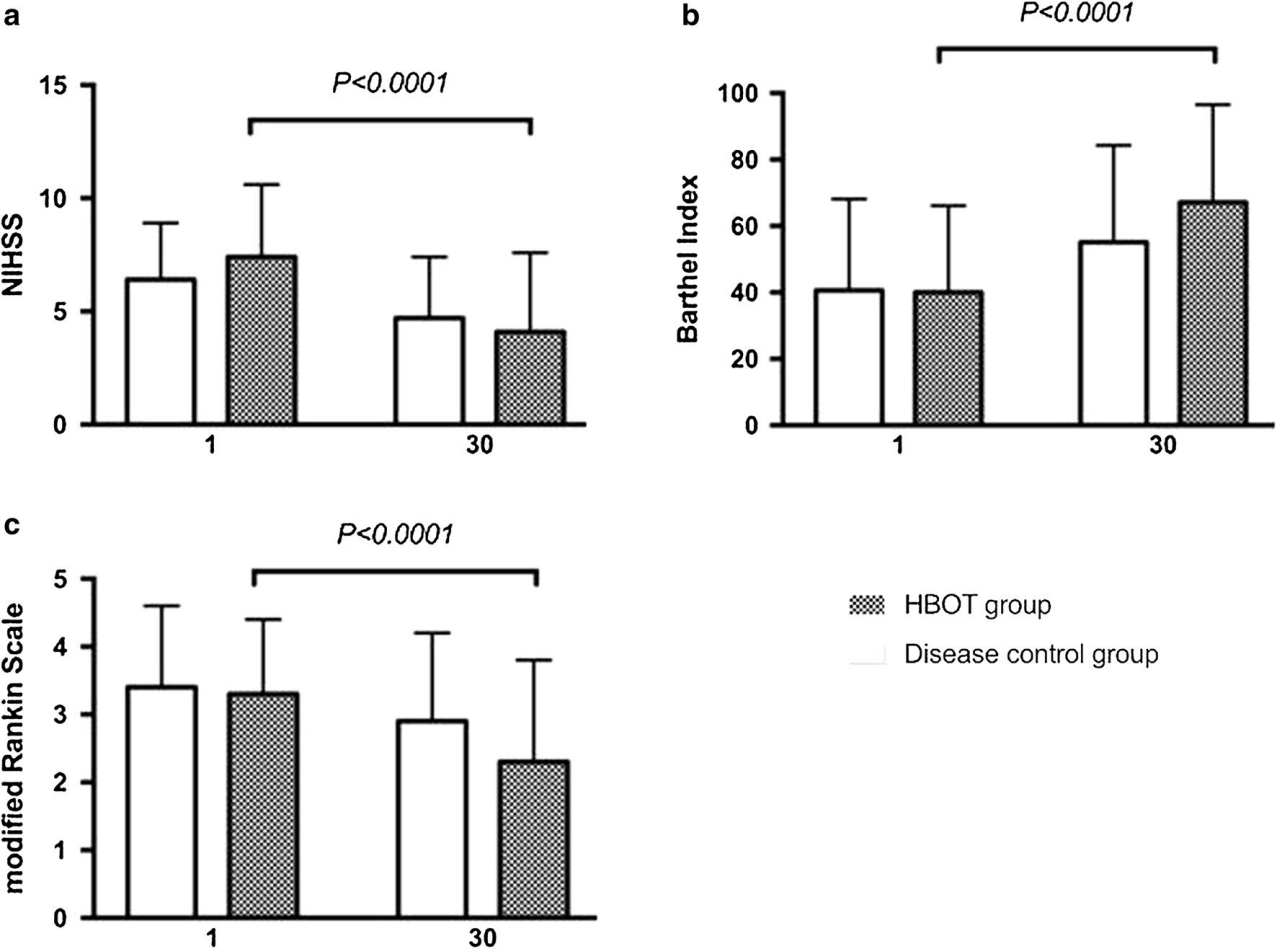
Changes (baseline and 1-month follow-up) in biomarker levels in the different patient subgroups (HBOT group and disease control) were compared using the paired-t test. **a** NIHSS, **b** Barthel index (%), **c** modified Rankin Scale

Changes in clinical scores at pre- and post-HBOT showed significant changes in NIHSS (*p *< 0.0001), BI (*p *< 0.0001), and MRS (*p *< 0.0001) in the HBOT group as well as significant changes in NIHSS (*p *= 0.001), BI (*p *= 0.013), and MRS (*p *= 0.049) in the disease control group (Table 3 and Fig. 2).

To exclude the possible effects of sex and age on clinical scores, the hypothesis that the clinical scores were equal between sexes and among ages was tested using ANCOVA. Univariate ANCOVA between the two treated groups at two different time points (baseline and postoperative follow-up) showed that NIHSS (*p *= 0.810), BI (*p *= 0.048), and MRS (*p *= 0.325) were statistically different, whereas NIHSS (*p *= 0.810) and MRS (*p *= 0.325) were not.

## Discussion

To date, this is the first study to show serial changes in circulating EPC and clinical scores in non-cardioembolic stroke patients following HBOT. This study also confirmed the hypothesis that HBOT not only improves clinical outcomes (BI), it increases the number of circulating EPCs [both KDR^+^/CD34^+^ (%) and CD133^+^/CD34^+^ (%)].

The present study examined changes in biomarkers on EPC and clinical scores in non-cardioembolic stroke patients before and after HBOT. There were three major findings. First, the numbers of KDR^+^/CD34^+^ (%) in the HBOT group following HBOT significantly increased, whereas the numbers of CD133^+^/CD34^+^ (%) also showed a tendency to increase but without statistical significance. Second, the changes of KDR^+^/CD34^+^EPCs (%) numbers were positively correlated with the changes in clinical outcomes scores (BI, NIHSS, and MRS) in the HBOT group. Third, the mean hs-CRP level significantly decreased at post-HBOT follow-up in the HBOT group.

Several studies using variations of basic EPC culture methods and flow cytometry techniques demonstrated that changes in circulatory EPC concentrations affected clinical outcomes of acute ischemic stroke and found that increased numbers of EPCs at the acute phase of acute ischemic stroke can improve clinical outcomes [7, 15, 22]. The present study showed that HBOT for acute non-cardioembolic stroke increased the number of circulating EPCs following HBOT (1 month after stroke).

Hs-CRP is a well-known inflammatory marker that is associated with ischemic stroke outcomes [23–25]. The present study showed that HBOT for acute non-cardioembolic stroke can statistically decrease hs-CRP levels following HBOT (1 month after stroke). Although an animal study demonstrated that HBOT can attenuate brain inflammation, ischemia, and oxidative damage [11, 12], HBOT in stroke in humans is still not sufficiently evidence-based due to insufficient numbers of randomized double-blind controlled clinical studies. Our study demonstrated decreased TBARS and increased thiol levels in both groups (HBOT and disease control groups). Several mechanisms are implicated in HBOT improving clinical outcomes (BI) in acute non-cardioembolic stroke, including: (1) the induction of EPCs could be an important mechanism responsible for HBOT inducing neurogenesis and brain angiogenesis; and (2) HBOT could alleviate oxidative stress and reduce inflammatory reactions.

The present study has several limitations. First, we only enrolled patients with first-event non-cardioembolic stroke with mild-to-moderate neurological deficits, while those who were comatose or considered unlikely to survive for > 1 week and those with pre-existing neurological conditions were excluded. Thus, an uncertainty is present in assessing the effects of HBOT in patients with cerebral infarctions who were not selected. Second, our study was not a double-blind randomized study, although the baseline neurological conditions were similar (e.g., NIHSS). Third, appropriate cell-surface markers specific for the identification of EPCs in the field of EPC research are lacking. Fourth, other drugs, including statins and angiotensin II receptor antagonists, may influence the number of circulating EPCs in patients with acute ischemic stroke [7]. Finally, the sample size was small; thus, prospective multicenter investigations with long-term follow-up are warranted to confirm the effects of HBOT on patients with acute ischemic stroke.

## Conclusions

Our study results suggest that HBOT can improve the short-term clinical outcomes and increase the number of circulating EPCs in patients with acute non-cardioembolic stroke.

## Abbreviations

EPCs: endothelial progenitor cells
HBOT: hyperbaric oxygen therapy
NIHSS: National Institutes of Health Stroke Scale
BI: Barthel index
MRS: modified Rankin Scale
ROS: reactive oxygen species
MRI: magnetic resonance imaging
HDL-C: high-density lipoprotein cholesterol
LDL-C: low-density lipoprotein cholesterol
hs-CRP: high-sensitivity C-reactive protein
TBARS: thiobarbituric acid-reactive substances
ANCOVA: analysis of covariance
